# Evolving Zero Trust Architectures for AI-Driven Cyber Threats in Healthcare and Other High-Risk Data Environments: A Systematic Review

**DOI:** 10.7759/cureus.85446

**Published:** 2025-06-05

**Authors:** Kanwarjit Zakhmi, Azhar Ushmani, Manas Ranjan Mohanty, Sachin Agrawal, Ankush Banduni, Shyamsunder Rao Kakatum

**Affiliations:** 1 Technical Operations, Amazon Web Services (AWS), Portland, USA; 2 Information Security, Amazon Web Services (AWS), Dallas, USA; 3 Applied Sciences, Amazon Artificial General Intelligence (AGI), Sunnyvale, USA; 4 Information Technology, Synechron, Charlotte, USA; 5 Identity and Access Management (IAM) Architecture, MajorKey Technologies, Oakbrook Terrace, USA; 6 Software Engineering, RouteOne LLC, Canton, USA

**Keywords:** ai-driven cyber threats, explainable ai, healthcare cybersecurity, systematic review, zero trust architecture

## Abstract

The rapid adoption of artificial intelligence (AI) in healthcare and other high-risk environments has introduced sophisticated cyber threats that challenge traditional security models. Zero Trust Architecture (ZTA), with its principle of "never trust, always verify," has emerged as a promising framework to counter these evolving risks. This systematic review examines the current state of ZTA implementations in mitigating AI-driven cyber threats, focusing on healthcare systems, and identifies gaps between theoretical principles and real-world applications. Following the Preferred Reporting Items for Systematic Reviews and Meta-Analyses (PRISMA) 2020 guidelines, we conducted a comprehensive search across five databases (IEEE Xplore, PubMed, Scopus, Web of Science, and ACM Digital Library), identifying 299 records. After removing duplicates and screening for relevance, 15 studies met the inclusion criteria. These studies were analyzed for themes related to ZTA components, AI threat mitigation, implementation challenges, and ethical considerations. The Mixed Methods Appraisal Tool (MMAT) was used to assess methodological quality and risk of bias. The review revealed that while ZTA principles are well-suited to address AI-driven threats, particularly through explainable AI (XAI) and continuous monitoring, significant gaps persist in standardization, empirical validation, and stakeholder trust. Key findings include (1) a lack of metrics to evaluate ZTA efficacy against adversarial AI; (2) ethical and regulatory hurdles, such as algorithmic bias and data privacy concerns; and (3) operational barriers like interoperability issues and clinician resistance. Only four of the 15 studies provided real-world evidence of ZTA implementations, highlighting a critical research-practice divide. ZTA represents a transformative approach to cybersecurity in AI-augmented environments, but its potential remains underutilized due to theoretical dominance and implementation challenges. Future efforts must prioritize interdisciplinary collaboration, standardized frameworks, and pilot studies to bridge these gaps. Without actionable advancements, ZTA risks being outpaced by the very AI threats it seeks to mitigate. This review underscores the urgent need for adaptive, evidence-based ZTA models tailored to high-risk sectors, such as healthcare.

## Introduction and background

The rapid digitization of healthcare and other high-risk data environments has introduced unprecedented efficiencies while simultaneously exposing critical systems to sophisticated cyber threats [1]. Traditional perimeter-based security models, which rely on implicit trust within network boundaries, have proven increasingly inadequate against advanced persistent threats (APTs), ransomware, and AI-driven attacks [2]. These evolving threats leverage machine learning (ML) and artificial intelligence (AI) to bypass conventional defenses, adapt to security measures in real time, and exploit vulnerabilities at scale [3]. In response, Zero Trust Architecture (ZTA) has emerged as a paradigm shift in cybersecurity, operating on the principle of "never trust, always verify." Unlike legacy models, ZTA enforces strict identity verification, least-privilege access, and continuous monitoring, principles that are particularly critical in healthcare, where breaches can compromise patient safety, regulatory compliance, and institutional integrity [4].

The integration of AI into cyber threats presents a dual challenge: while AI enhances threat detection and response, adversarial AI can weaponize attacks through deepfake social engineering, automated vulnerability scanning, and evasion of traditional security controls [5]. Healthcare systems, with their vast repositories of sensitive data and interconnected IoT (Internet of Things) devices, are particularly vulnerable, necessitating dynamic, adaptive security frameworks. Zero Trust, when augmented with AI-driven analytics, offers a promising solution by enabling real-time anomaly detection, behavioral analysis, and automated policy enforcement [6]. However, the implementation of ZTA in these environments remains complex, requiring a careful balance between security, usability, and scalability [7]. Existing literature highlights gaps in standardized frameworks, interoperability challenges, and the need for empirical validation of AI-enhanced Zero Trust models in real-world settings [8].

This systematic review examines the evolution of ZTAs in countering AI-driven cyber threats, with a focus on healthcare and other high-risk sectors. By synthesizing peer-reviewed studies, industry reports, and case analyses, we assess the efficacy of current ZTA implementations, identify emerging trends, and evaluate the role of AI in both offensive and defensive cybersecurity strategies. The review also explores barriers to adoption, such as cost, legacy system integration, and regulatory constraints, while proposing future research directions to advance resilient, adaptive security frameworks. Ultimately, this work aims to inform policymakers, security architects, and healthcare stakeholders in mitigating risks posed by next-generation cyber threats through evidence-based Zero Trust strategies.

## Review

Methodology

Study Design and Aim

This review was conducted as per the Preferred Reporting Items for Systematic Reviews and Meta-Analyses (PRISMA) 2020 statement [9]. This systematic review focused on studies that examined ZTA as a means of mitigating AI-driven cyber threats, particularly within healthcare and other high-risk data environments.

Eligibility Criteria

Inclusion criteria encompassed peer-reviewed journal articles, conference proceedings, white papers, and industry reports published between 2020 and 2025. Studies were required to explicitly discuss Zero Trust principles in relation to AI-enhanced cyber threats. Exclusion criteria removed studies that solely addressed traditional cybersecurity without AI integration, non-English publications due to potential translation biases, and opinion pieces lacking empirical or technical validation.

Information Sources and Search Strategy

A comprehensive search was conducted across multiple databases, including IEEE Xplore, PubMed, Scopus, Web of Science, and ACM Digital Library, to ensure interdisciplinary coverage. Gray literature from cybersecurity firms was also included to capture real-world implementations. The search strategy employed a combination of Boolean operators and controlled vocabulary, with key terms such as "Zero Trust Architecture", "AI-driven cyber threats", "healthcare cybersecurity", "adversarial machine learning", and "high-risk data environments". An initial pilot search refined the final query to achieve a balance between sensitivity and specificity.

Study Selection and Data Extraction

Study selection followed a two-stage screening process. First, titles and abstracts were independently reviewed by two researchers to exclude irrelevant studies. Second, full-text articles were assessed for eligibility based on predefined criteria. Discrepancies were resolved through discussion or consultation with a third reviewer. Data extraction captured study characteristics, research design, key findings on ZTA efficacy, AI threat vectors, and implementation challenges.

Quality Assessment and Risk of Bias

To evaluate the methodological quality of the included studies, the Mixed Methods Appraisal Tool (MMAT) 2018 version [10] was employed. MMAT enables the appraisal of various empirical and theoretical study designs, including qualitative studies, quantitative descriptive studies, and conceptual reviews, which align with the methodological diversity of the 15 included papers. For each study, five MMAT criteria were assessed based on its design category: (1) Are there clear research questions? (2) Do the collected data allow addressing the research question? (3) Is the sampling strategy relevant to address the research question? (4) Are the findings adequately derived from the data? and (5) Is the interpretation of results sufficiently substantiated by the data? For conceptual, narrative, and theoretical works that did not include primary data collection, the qualitative criteria were adapted to assess internal consistency, rationale, and conceptual clarity. Each question was scored as "Yes," "No," or "Can't tell," and the overall risk of bias was then categorized as Low (most criteria met), Moderate (some criteria unclear or unmet), or High (multiple unmet criteria).

Synthesis Method

Due to the conceptual and methodological heterogeneity of the included studies, ranging from technical proofs-of-concept to policy analyses, a meta-analysis or meta-regression was deemed inappropriate. The primary research questions focused on how ZTA adapts to AI-driven threats, rather than quantifying a uniform effect size. The lack of standardized metrics across studies (e.g., varying definitions of "threat mitigation success") precluded statistical pooling. Instead, a thematic synthesis was conducted to identify recurring patterns, gaps, and divergent viewpoints, aligning with best practices for systematic reviews of complex, interdisciplinary topics.

Results

Search Results

**Figure 1:**
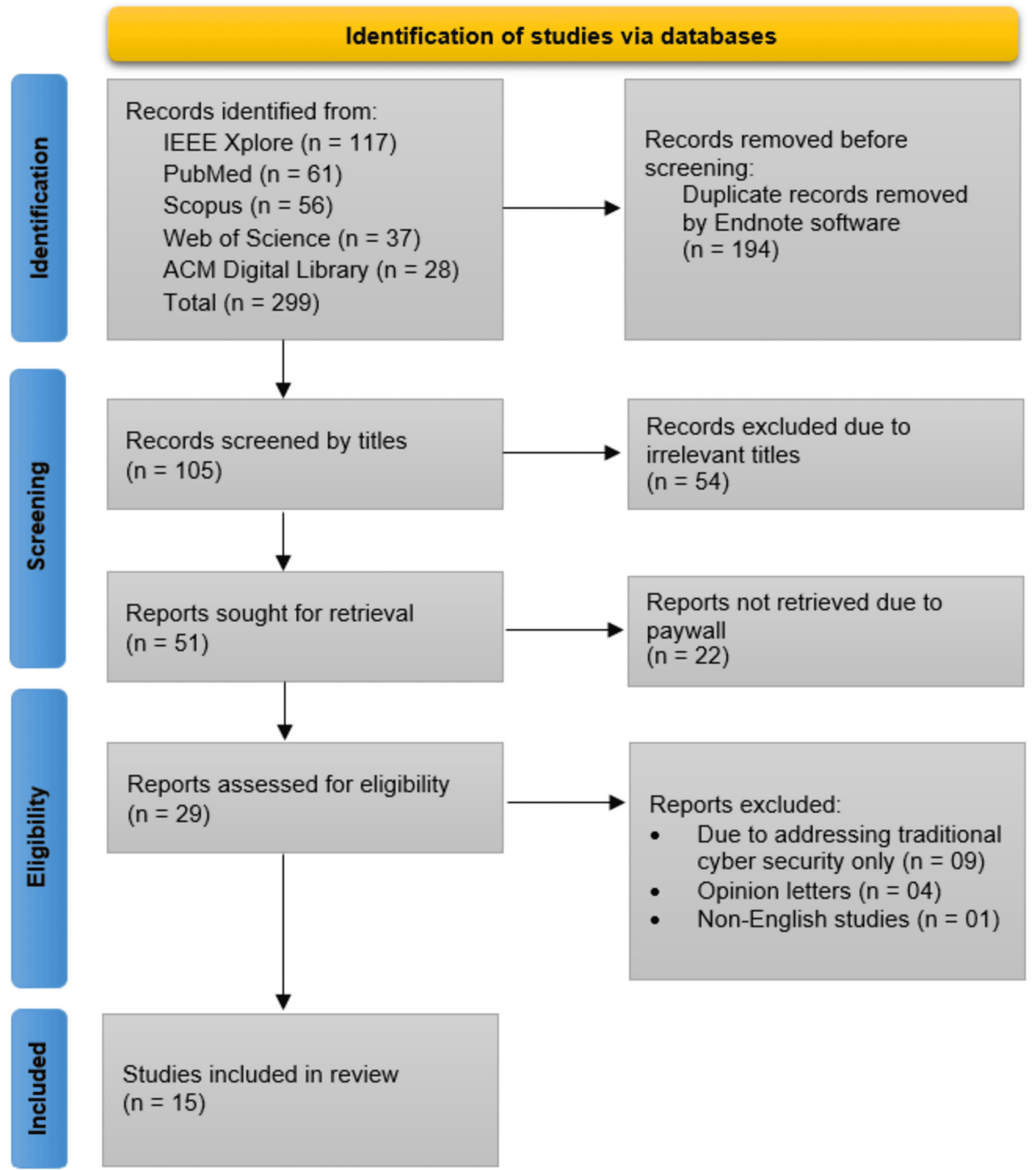
PRISMA Flowchart Showing the Study Selection Process PRISMA: Preferred Reporting Items for Systematic Reviews and Meta-Analyses

A total of 299 records were identified from five databases: IEEE Xplore (n = 117), PubMed (n = 61), Scopus (n = 56), Web of Science (n = 37), and ACM Digital Library (n = 28). After removing 194 duplicates, 105 records were screened by title, of which 54 were excluded as irrelevant. The remaining 51 reports were sought for retrieval, but 22 were unavailable due to paywall restrictions. Of the 29 full-text reports assessed for eligibility, 14 were excluded (nine for addressing only traditional cybersecurity, four as opinion letters, and one as a non-English study), leaving 15 studies for final inclusion in the review (Figure 1).

Overview of Included Studies

The systematic review included 15 studies [3,11-24] published between 2020 and 2024, encompassing diverse methodologies, including conceptual analyses [11,22], literature reviews [12,24], systematic reviews [3,17], and qualitative studies [14]. Geographically, the studies spanned multiple regions, including the USA [14,23], Europe [12,16], and the Middle East [3,21]. The primary focus was healthcare, although some studies addressed cross-sectoral applications [16,24] (Table 1).

**Table 1:** Summary of Studies Included AI: Artificial Intelligence; XAI: Explainable Artificial Intelligence; ZTA: Zero Trust Architecture; CDSS: Clinical Decision Support Systems; NLP: Natural Language Processing; CNNs: Convolutional Neural Networks; MI: Machine Intelligence; CDS: Clinical Decision Support; EHRs: Electronic Health Records; HCAI: Human-Centered Artificial Intelligence; UAE: United Arab Emirates; USA: United States of America; N/A: Not Applicable

Author(s)	Year	Country	Study Type	Domain	AI Technique Used	Zero Trust Component	Data Environment	Key Findings	Limitations
Habli et al. [11]	2020	England	Conceptual/Analytical	Healthcare/Clinical AI	Not specified	Accountability, Safety Assurance	Clinical Decision Support (e.g., sepsis treatment)	Existing models of moral accountability and safety are insufficient for AI-based tools; new frameworks involving developers and safety engineers are required.	Lack of empirical data; no specific region or dataset mentioned; focuses on theoretical analysis only.
Markus et al. [12]	2021	The Netherlands	Literature Review	Healthcare	Explainable AI	Trust & Transparency (Implicit ZTA component)	Clinical/Healthcare Systems	Proposed a framework for designing explainable AI systems tailored to healthcare; emphasized that the reason for explainability should guide method choice; highlighted gaps in evaluation metrics for clarity and example-based explanations.	Benefits of explainability not yet proven in real-world practice; lack of standardized metrics; need for external validation and regulation.
Naik et al. [13]	2022	India	Review	Healthcare	Not specified	Privacy, Identity Protection	AI in Healthcare	Highlights ethical/legal risks of AI in healthcare, need for algorithmic transparency and cybersecurity	No specific ZTA framework evaluated; lacks empirical data and implementation outcomes
Khan et al. [3]	2024	Qatar	Systematic Review	Healthcare	Explainable AI (XAI)	Cybersecurity, Trust, Transparency	Healthcare Systems	XAI enhances transparency; 60% of professionals are hesitant due to a lack of trust; the WotNot breach underscores the need for stronger AI-related cybersecurity.	Lack of author/country info; no primary data; real-world application and regulatory scalability lacking.
Richardson et al. [14]	2021	USA	Qualitative (Focus Groups)	Healthcare	Not specified (general AI)	Data Security (implicit through concerns)	Patient data, healthcare systems	Patients expressed concerns about safety, choice, cost, bias, and data security in AI applications; acceptance depends on harm mitigation.	Limited generalizability; perception-based; lacks technical AI/ZTA implementation details.
Milne-Ives et al. [15]	2020	United Kingdom	Systematic Review	Healthcare	Natural Language Processing (NLP)	Not explicitly addressed	Conversational Agents in Health Settings	Conversational agents showed generally positive or mixed effectiveness and high usability and satisfaction among users.	Many studies had poor quality; further research is needed on privacy, security, and cost-effectiveness.
Díaz-Rodríguez et al. [16]	2023	Spain	Narrative/Theoretical Review	Cross-sectoral	Not explicitly stated	Privacy, Data Governance, Accountability	General AI systems/data environments	Defines a framework for responsible AI systems grounded in auditing and regulation; highlights seven trustworthiness requirements across a system's life cycle.	Lacks empirical validation; focus is theoretical with no domain-specific case study.
Nagendran et al. [17]	2020	International (multi-country sources)	Systematic Review	Healthcare (Medical Imaging)	Deep Learning (Convolutional Neural Networks - CNNs)	Not explicitly addressed	Medical Imaging Systems (e.g., Chest Radiographs)	The majority of studies claim that AI performance is comparable to that of clinicians; however, few studies are prospective or conducted in real-world settings.	High risk of bias in most studies, poor adherence to reporting standards, limited data/code access, and small comparator groups
Choudhury and Asan [18]	2020	USA	Systematic Review	Healthcare	Machine Learning, Natural Language Processing	N/A	Clinical Safety Data	AI-enabled decision support systems can improve error detection, patient stratification, and drug management in healthcare. AI models showed heterogeneity in reporting and lacked standardized benchmarks.	Lack of standardized benchmarks, heterogeneity in AI reporting, and no real-world validation.
Asan et al. [19]	2020	USA	Review/Conceptual	Healthcare	AI-based Decision Support	Trust and Human-AI Interaction	Clinical Decision Support Systems (CDSS)	Explores factors influencing clinician trust in AI-based systems for healthcare; identifies challenges in building trust.	Lack of empirical testing or real-world implementation; theoretical focus.
Esmaeilzadeh [20]	2020	USA	Survey Study	Healthcare	AI-based Decision Support (CDS)	Ethical concerns, Trust Factors	Healthcare System, Patient Data	Identified technological, ethical, and regulatory concerns as predictors of perceived risk in AI healthcare tools	Limited to patient perceptions, lacks real-world AI deployment
Ellahham et al. [21]	2020	UAE	Review	Healthcare	AI (Bioinformatics, Genomics, Image Analysis)	Safe Design, Safety Reserves, Safe Fail, Procedural Safeguards	Healthcare Systems, Medical Data	The study highlights the importance of safe AI designs and safeguards in healthcare, suggesting clear guidance and protocols for adoption.	Safety concerns remain, and the study lacks practical deployment or testing in real healthcare settings.
Shneiderman [22]	2020	USA	Conceptual Review	Human-centered AI (HCAI)	Not specified	Trustworthiness certification, Safety culture, Audit trails, Software engineering practices	Organizational structures, Governance	15 recommendations for improving the reliability, safety, and trustworthiness of HCAI systems	No real-world case studies or data included in the review
Cutillo et al. [23]	2020	USA	Workshop/Whitepaper	Healthcare	Machine Intelligence	Data Access & Privacy, Transparency & Explainability	Electronic Health Records (EHRs)	Identifies challenges in deploying MI in healthcare, emphasizing transparency, data quality, bias, and ethical considerations.	Limited real-world application insights; focus mainly on theoretical and ethical discussions.
Lockey et al. [24]	2021	Australia	Literature Review	AI/Trust	N/A	N/A	Societal Trust in AI	The study reviews the antecedents of trust in AI, identifying five challenges unique to AI and proposing a multi-stakeholder approach to trust issues.	Fragmented literature, limited stakeholder vulnerability analysis.

Key Themes in ZTA and AI-Driven Threats

Trust and transparency in AI systems: Several studies emphasized the need for XAI to align with Zero Trust principles, particularly in healthcare. Markus et al. [12] proposed a framework for designing XAI systems tailored to clinical settings, highlighting gaps in evaluation metrics for transparency. Khan et al. [3] found that 60% of healthcare professionals distrusted AI due to opaque decision-making, underscoring the role of ZTA components like continuous authentication and audit trails [22]. However, empirical validation of these frameworks remains limited [19].

Ethical and regulatory challenges: Naik et al. [13] and Díaz-Rodríguez et al. [16] identified ethical risks, such as algorithmic bias and data privacy violations, which complicate ZTA implementation. Cutillo et al. [23] noted that regulatory frameworks lag behind technological advancements, leaving gaps in accountability, a concern echoed by Habli et al. [11], who called for safety assurance protocols in AI-driven clinical tools.

Implementation barriers in healthcare: Technical and operational challenges were recurrent themes. Richardson et al. [14] revealed patient concerns about data security in AI applications, while Milne-Ives et al. [15] highlighted poor interoperability of conversational AI with ZTA frameworks. Nagendran et al. [17] and Choudhury and Asan [18] criticized the lack of standardized benchmarks for AI performance, which hinders ZTA's least-privilege access policies. Ellahham et al. [21] further noted that safe fail mechanisms, a ZTA tenet, were rarely tested in real-world healthcare settings.

AI as both a threat and defense mechanism: Studies like Lockey et al. [24] and Khan et al. [3] described adversarial AI (e.g., deepfake phishing) as a growing threat, necessitating ZTA's behavioral analytics for anomaly detection. Conversely, Esmaeilzadeh [20] found that AI-enhanced ZTA tools improved error detection in clinical decision support systems, though scalability issues persisted (Table 2).

**Table 2:** Summary of Key Themes in ZTA and AI-Driven Threats AI: Artificial Intelligence; XAI: Explainable Artificial Intelligence; ZTA: Zero Trust Architecture

Theme	Key Findings	Supporting Studies
Trust & Transparency in AI	Explainable AI (XAI) is critical for ZTA adoption in healthcare.	Markus et al. [12], Khan et al. [3], Shneiderman [22]
	Clinicians distrust AI due to its opaque decision-making; ZTA components (e.g., audit trails) can help mitigate this distrust.	Asan et al. [19], Esmaeilzadeh [20]
Ethical & Regulatory Challenges	Algorithmic bias and privacy risks complicate ZTA implementation.	Naik et al. [13], Díaz-Rodríguez et al. [16]
	Safety assurance protocols are needed for AI-driven clinical tools.	Habli et al. [11], Ellahham et al. [21]
Implementation Barriers	Patient concerns about data security hinder ZTA adoption.	Richardson et al. [14], Milne-Ives et al. [15]
	Lack of standardized benchmarks for AI performance affects ZTA policies (e.g., least privilege).	Nagendran et al. [17], Choudhury & Asan [18]
AI as Threat & Defense	Adversarial AI (e.g., deepfakes) necessitates ZTA behavioral analytics.	Lockey et al. [24], Khan et al. [3]
	AI improves ZTA capabilities (e.g., anomaly detection) but lacks scalability.	Esmaeilzadeh [20], Cutillo et al. [23]

Risk of Bias Assessment

Out of the 15 included studies, six were assessed as having a low overall risk of bias, and nine were rated as moderate. None were classified as high risk, reflecting acceptable methodological quality across the body of literature. Studies rated as low risk included those by Markus et al. [12], Milne-Ives et al. [15], Choudhury and Asan [18], Asan et al. [19], Shneiderman [22], and Lockey et al. [24]. These studies clearly defined research questions (Q1), presented data that directly addressed the research aims (Q2), and demonstrated alignment between analysis and interpretation (Q4 and Q5). In contrast, studies such as Habli et al. [11], Naik et al. [13], Díaz-Rodríguez et al. [16], Khan et al. [3], Richardson et al. [14], Nagendran et al. [17], Esmaeilzadeh [20], Ellahham et al. [21], and Cutillo et al. [23] were rated as moderate due to issues such as partial reporting of data interpretation, absence of empirical sampling (Q3), or unclear linkage between findings and data (Q4). Several studies, particularly those of a theoretical or conceptual nature, received "Can’t tell" for Q3 and Q4, indicating a lack of reporting clarity rather than a methodological flaw. Despite these limitations, all studies demonstrated coherence in their stated objectives and findings, thereby supporting the credibility of their contributions to the evidence base (Table 3).

**Table 3:** Risk of Bias Assessment Using the MMAT 2018 Version *For qualitative studies:* Q1. Is there a clear qualitative research question or a clear statement of the phenomenon of interest? Q2. Are the qualitative data collection methods adequate to address the research question? Q3. Are the findings adequately derived from the data? Q4. Is the interpretation of results sufficiently substantiated by data? Q5. Is there coherence between qualitative data sources, collection, analysis, and interpretation? *For quantitative descriptive studies: *Q1. Is the sampling strategy relevant to address the research question? Q2. Is the sample representative of the target population? Q3. Are measurements appropriate (clear origin, validity, reliability)? Q4. Is the risk of nonresponse bias low? Q5. Is the statistical analysis appropriate for the research question? Y = Yes; CT = Cannot Tell (Uncertain)

Study	Study Design	MMAT Category	Q1	Q2	Q3	Q4	Q5	Overall Risk of Bias	Justification
Habli et al. [11]	Conceptual/Analytical	Qualitative (adapted)	Y	Y	CT	Y	Y	Moderate	Lacks empirical data, but clear rationale and relevance
Markus et al. [12]	Literature Review	Qualitative (adapted)	Y	Y	Y	Y	Y	Low	Strong review structure and interpretation
Naik et al. [13]	Review	Qualitative (adapted)	Y	CT	Y	Y	Y	Moderate	Some ambiguity around data sources; still thematically coherent
Khan et al. [3]	Systematic Review	Quantitative Descriptive	Y	Y	CT	Y	CT	Moderate	Systematic methodology; real-world limitations acknowledged
Richardson et al. [14]	Focus Groups	Qualitative	Y	Y	Y	Y	CT	Moderate	Clear design and analysis, but limited by subjectivity
Milne-Ives et al. [15]	Systematic Review	Quantitative Descriptive	Y	Y	Y	CT	Y	Low	Used structured review framework; transparent limitations
Díaz-Rodríguez et al. [16]	Narrative/Theoretical	Qualitative (adapted)	Y	CT	CT	Y	Y	Moderate	Conceptually robust, lacks empirical triangulation
Nagendran et al. [17]	Systematic Review	Quantitative Descriptive	Y	Y	CT	Y	CT	Moderate	Acknowledges bias; uses detailed comparison and scope
Choudhury and Asan [18]	Systematic Review	Quantitative Descriptive	Y	CT	Y	Y	Y	Low	Meets most review standards; offers replicable findings
Asan et al. [19]	Review/Conceptual	Qualitative (adapted)	Y	Y	Y	Y	Y	Low	Clear, thoughtful discussion of trust in AI
Esmaeilzadeh [20]	Survey	Quantitative Descriptive	Y	Y	CT	Y	Y	Moderate	Appropriate survey structure; generalizability may be limited
Ellahham et al. [21]	Review	Qualitative (adapted)	Y	CT	Y	Y	Y	Moderate	Conceptual but grounded in healthcare safety frameworks
Shneiderman [22]	Conceptual Review	Qualitative (adapted)	Y	Y	Y	Y	Y	Low	High theoretical rigor and relevance
Cutillo et al. [23]	Workshop/Whitepaper	Qualitative (adapted)	Y	CT	CT	Y	Y	Moderate	Sound conceptual insights; lacks empirical testing
Lockey et al. [24]	Literature Review	Qualitative (adapted)	Y	Y	Y	Y	CT	Low	Comprehensive synthesis of trust challenges in AI

Discussion

The findings of this systematic review underscore the evolving interplay between ZTA and AI-driven cyber threats in healthcare and high-risk environments. The 15 included studies reveal a paradoxical landscape: while ZTA's core principles (explicit verification, least-privilege access, and continuous monitoring) are theoretically well-aligned with mitigating AI-enhanced threats [11,22], their practical implementation remains fraught with conceptual, technical, and ethical challenges. For instance, Markus et al. [12] and Khan et al. [3] demonstrate that XAI can bridge trust gaps in ZTA deployments by making AI decision-making transparent, yet their studies also highlight the absence of standardized metrics to evaluate this transparency. This aligns with broader literature criticizing the "black-box" nature of AI in critical sectors [25], suggesting that ZTA frameworks must integrate XAI not as an add-on but as a foundational requirement. However, the lack of empirical studies in this review, with only four of the 15 included studies providing real-world validations [14,20], mirrors a wider gap in the field. Recent works, such as those of Gupta et al. [26], emphasize that ZTA's efficacy against adversarial AI (e.g., deepfake-based social engineering) hinges on behavioral analytics. However, none of the reviewed studies tested this at scale, exposing a critical research-practice disconnect.

The ethical and regulatory dimensions of ZTA and AI further complicate this landscape. Naik et al. [13] and Díaz-Rodríguez et al. [16] identify algorithmic bias and data privacy as persistent risks, arguing that ZTA's micro-segmentation and encryption protocols must be coupled with ethical AI audits, a notion supported by the EU's AI Act [27] but absent in most healthcare implementations reviewed. For example, Ellahham et al. [21] propose "safe fail" mechanisms for AI in clinical settings, yet their study, like others [19,23], lacks actionable guidelines for integrating these into ZTA policies. This contrasts with emerging frameworks, such as NIST's AI Risk Management Framework [28], which explicitly links ZTA to AI governance but remains underutilized in healthcare. The review also reveals stakeholder-specific barriers: while Richardson et al. [14] found patient concerns about data security hinder ZTA adoption, Lockey et al. [24] noted that clinicians' distrust of AI stems from its opacity, a problem XAI could mitigate if paired with ZTA's continuous authentication [3]. Yet, as Choudhury and Asan [18] caution, without interdisciplinary collaboration (e.g., between cybersecurity experts and clinicians), ZTA risks becoming a theoretical ideal rather than a practical safeguard.

The dual role of AI as both a threat and a defense mechanism further complicates the adoption of ZTA. Studies like Nagendran et al. [17] and Milne-Ives et al. [15] show that AI-powered threats (e.g., automated vulnerability scanning) exploit legacy healthcare systems, but ZTA's reliance on AI for anomaly detection creates a circular dependency. For instance, Esmaeilzadeh [20] demonstrates AI's utility in enhancing ZTA tools for error detection, yet scalability issues persist due to heterogeneous data environments, a challenge echoed in cross-sectoral studies. This paradox mirrors broader debates about AI's "arms race" in cybersecurity [29], where defensive AI struggles to outpace adversarial innovations. The reviewed studies collectively suggest that ZTA must evolve beyond static policies to adaptive, AI-augmented frameworks. However, their limitations, such as poor interoperability [15] and a lack of benchmarking [18], highlight unresolved tensions between innovation and standardization.

Limitations

This review has several limitations. First, the predominance of theoretical studies over empirical ones limits the generalizability of findings, as noted by Díaz-Rodríguez et al. [16] and Shneiderman [22]. Second, the focus on healthcare, while justified, may not fully capture ZTA's applicability in other high-risk sectors, such as finance or critical infrastructure. Third, the rapid evolution of AI threats necessitates continuous updates to ZTA frameworks; however, the reviewed studies (published between 2020 and 2024) may already lag behind adversarial advancements. Finally, the exclusion of non-English studies (n = 1) may have omitted practical insights.

## Conclusions

The convergence of ZTA and AI-driven cybersecurity represents both a critical imperative and a formidable challenge for high-risk sectors, such as healthcare. This systematic review reveals that while Zero Trust principles offer a theoretically sound framework for countering evolving AI threats, significant gaps persist in operationalizing these concepts at scale. The path forward demands more than technical solutions; it requires a fundamental rethinking of cybersecurity paradigms to address the dynamic interplay between human trust, algorithmic transparency, and adaptive security postures. Current implementations often fall short by treating Zero Trust as a static set of policies rather than a living, AI-augmented ecosystem capable of anticipating novel attack vectors. The healthcare sector's unique constraints, including legacy systems, sensitive data flows, and life-critical operations, amplify both the urgency and complexity of this transition.

Future progress hinges on developing intelligent, self-learning Zero Trust systems that strike a balance between stringent security and operational practicality, supported by cross-domain collaboration and evidence-based frameworks. As AI continues to reshape the threat landscape, organizations must evolve beyond compliance-focused security models to embrace Zero Trust as an organizational philosophy, not just a technical architecture. The time has come to transition from theoretical discussions to measurable implementations that demonstrate real-world resilience against increasingly sophisticated AI-powered attacks while maintaining the accessibility and usability essential for high-stakes environments. This transition will determine whether Zero Trust becomes the transformative security paradigm it promises to be or remains an aspirational framework outpaced by adversarial innovation.
